# ﻿Two new species of the purse-web spider genus *Atypus* Latreille, 1804 from China (Araneae, Atypidae)

**DOI:** 10.3897/zookeys.1229.143547

**Published:** 2025-02-27

**Authors:** Yangxin Li, Fan Li, Daiqin Li, Xin Xu

**Affiliations:** 1 College of Life Sciences, Hunan Normal University, Changsha 410081, Hunan Province, China Hunan Normal University Changsha China; 2 State Key Laboratory of Protein and Plant Gene Research, School of Life Sciences, Peking University, Beijing 100871, China Peking University Beijing China; 3 Centre for Behavioural Ecology and Evolution (CBEE), School of Life Sciences, Hubei University, 368 Youyi Road, Wuhan 430062, Hubei Province, China Hubei University Wuhan China

**Keywords:** Description, morphology, Mygalomorphae, taxonomy

## Abstract

Two new species of the purse-web spider genus *Atypus* Latreille, 1804, collected from China, are diagnosed and described based on the genital morphology of both sexes: *A.dawei***sp. nov.** (♂♀) and *A.liui***sp. nov.** (♂♀). These species are widespread in central China. *Atypusdawei***sp. nov.** is found in Anhui, Hunan, and Jiangxi provinces, while *A.liui***sp. nov.** is distributed across Anhui, Guangxi, Guizhou, Henan, Hubei, Hunan, and Jiangxi provinces.

## ﻿Introduction

**Figure 1.**
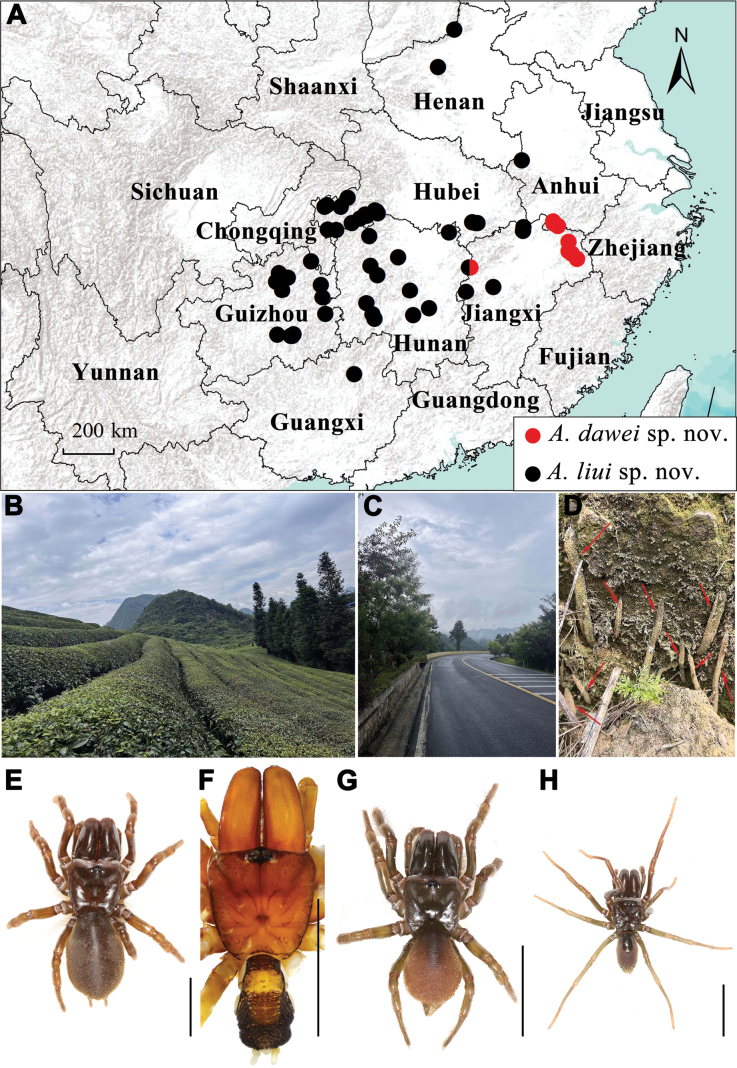
The collection localities (**A**), microhabitat (**B–D**) and general somatic morphology (**E–H**) of two new *Atypus* species. Red arrows show purse-webs. **E, G** female **F, H** male **E–F***Atypusdawei* sp. nov. **G–H***Atypusliui* sp. nov. **E** ATY-2023-212 **F** LY001 **G** ATY-2023-102F **H** ATY-2023-102M. Scale bars: 0.5 cm.

The purse-web spider family Atypidae Thorell, 1870, belonging to Mygalomorphae, currently comprises 56 valid species across three genera, *Atypus* Latreille, 1804, *Calommata* Lucas, 1837, and *Sphodros* Walckenaer, 1835 (World Spider Catalog 2025). Members of Atypidae live in burrows and typically construct tough, silken tubes, which may include subterranean and above-ground sections, as seen in *Atypus* (Fig. 1D) and *Sphodros*, or solely underground burrows with an open entrance, as in *Calommata* (Gertsch and Platnick 1980; Fourie et al. 2011; Li et al. 2022).

The genus *Atypus* currently includes 33 valid species across Asia, Europe, and North Africa, with one species, *A.karschi* Dönitz, 1887, introduced to North America by humans (Řezáč et al. 2022; World Spider Catalog 2025). Of these, 15 species are recorded from China, comprising 12 species described from both sexes and three species known from a single sex (World Spider Catalog 2025). Taxonomic revisions of Chinese *Atypus* were conducted by Zhu et al. (2006) resulted in the description of seven new species and the redescription of six known species. Over a decade later, only two additional *Atypus* species have been described from China (Li et al. 2018).

Morphologically, the genus *Atypus* can be distinguished from the other two atypid genera by the following characteristics: (1) males possess a sternum with marginal ridges; (2) the male palps feature a short, straight, spine-like embolus and a simple, distally expanded conductor; and (3) female genitalia exhibit bulbous or pyriform receptacula and two pore patches on the genital atrium (Schwendinger 1990; Zhu et al. 2006; Li et al. 2018).

In this study, we diagnose and describe two new *Atypus* species from China, based on the morphology of the male palp and female genitalia.

## ﻿Material and methods

All specimens were excavated alive from their underground silken tube (Fig. 1A). The right four legs of adult specimens were removed and preserved in absolute ethanol and stored at –80 °C for molecular analysis. The remaining parts of each specimen were preserved in 80% ethanol and stored as vouchers for morphological examination. All type and voucher specimens are deposited at the School of Life Sciences, Hubei University, Wuhan, Hubei Province, China.

Specimens were examined and dissected using an Olympus SZ61 stereomicroscope. The soft tissues of the female genitalia were removed and digested using a 10 mg/ml pancreatin solution (Biosharp Company, Hefei, Anhui, China) for at least 3 h at room temperature to remove excess soft tissue. Male palps and female genitalia were observed and photographed with a CCD digital camera mounted on an Olympus BX53 compound microscope. Compound-focused images were generated using Helicon Focus v. 6.7.1.

All measurements are provided in millimeters. Leg measurements are listed in the following order: total length (femur, patella, tibia, metatarsus, tarsus).

Abbreviations used:

**AL** abdomen length

**ALE** anterior lateral eyes

**ALS** anterior lateral spinnerets

**AME** anterior median eyes

**AW** abdomen width

**CL** carapace length

**CW** carapace width

**MOA** median ocular area

**PLE** posterior lateral eyes

**PLS** posterior lateral spinnerets

**PME** posterior median eyes

**PMS** posterior median spinnerets

**TL** total length

## ﻿Taxonomy

### ﻿Family Atypidae Thorell, 1870

**Genus *Atypus* Latreille, 1837**

#### ﻿ Atypus dawei

Taxon classificationAnimaliaAraneaeAtypidae

Li & Xu sp. nov.

2499F009-5528-5093-BBF9-07E0CD6283E3

https://zoobank.org/731CD2FF-CDD2-4539-9907-BED8B87A3A0A

Figs 1 , 2 , 3

##### Type material.

***Holotype***: China • ♂; Hunan Province, Liuyang City, Mount Dawei, Wuzhi Summit; 28.41°N, 114.11°E; alt. 1200 m; 13 May 2016; X. Xu leg.; LY001.

***Paratypes***: China • 2♀♀; same data as for holotype, alt. 740 m; 4–6 May 2022; ATY-2022-001, 002 – Jiangxi Province • 1♀; Shangrao City, Guangxin District, Zhengfang Town, Lou Village; 28.69°N, 117.90°E; alt. 131 m; 30 August 2023; ATY-2023-181 • 1♀; Shangrao City, Dexing City, Raoer Town, Damaoshan Scenic Area; 28.84°N, 117.72°E; alt. 185 m; 30 August 2023; ATY-2023-189 • 1♀; Shangrao City, Dexing City, Fenghuanghu Scenic Area; 28.92°N, 117.60°E; alt. 74 m; 31 August 2023; ATY-2023-192 • 2♀♀; Shangrao City, Wuyuan County, Xucun Town, Fenshui Village; 29.22°N, 117.61°E; alt. 84–92 m; 1 September 2023; ATY-2023-193, 195 • 2♀♀; Jingdezhen City, Fuliang County, Jinggongqiao Town; 29.70°N, 117.22°E; alt. 92 m; 2 September 2023; ATY-2023-204, 207 – Anhui Province, Chizhou City, Dongzhi County • 1♀; Muta Town, Hengshan Village; 29.79°N, 117.07°E; alt. 122 m; 2 September 2023; ATY-2023-209 • 2♀♀; Guangang Town, Huangjialong Village; 29.85°N, 117.05°E; alt. 125 m; 2 September 2023; X. Xu, Y. Zhang, Y.X. Li, J.Y. Yuan leg.; ATY-2023-212, 213.

##### Diagnosis.

**Figure 2.**
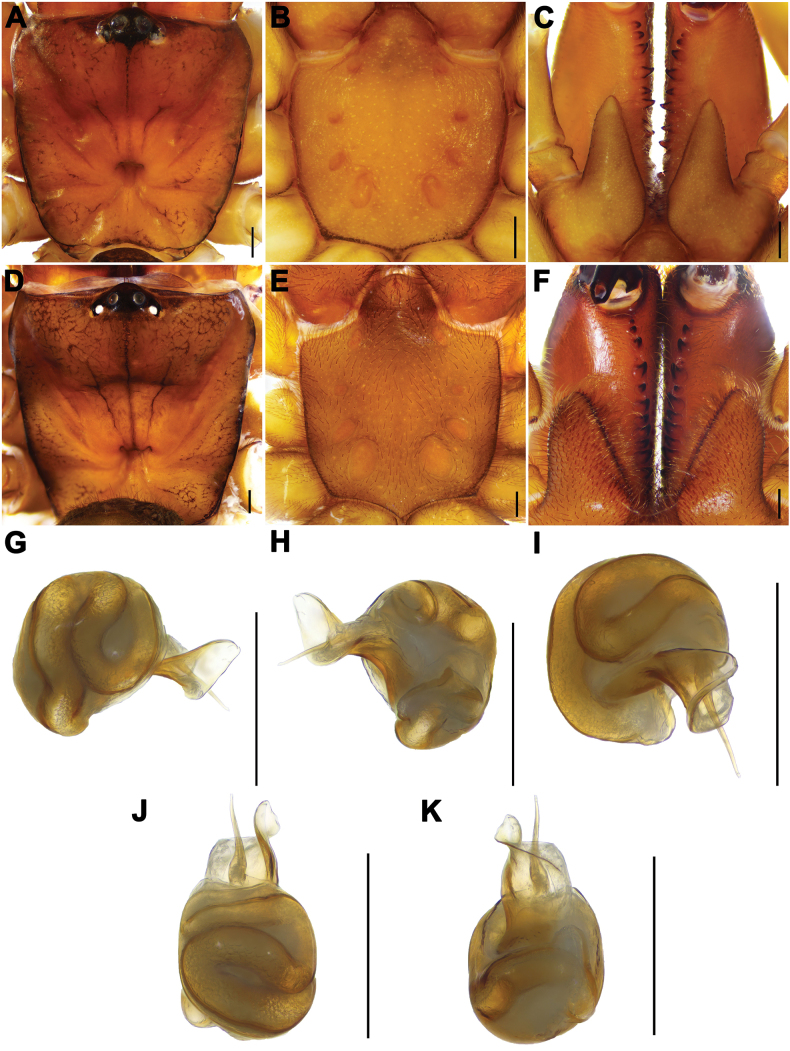
Male and female somatic structure and male genital anatomy of *Atypusdawei* sp. nov. **A–C** male (LY001) **D–F** female (ATY-2023-212) **A, D** carapace **B, E** sternum **C, F** chelicerae **G–K** left male palp (LY001) **G** prolateral view **H** retrolateral view **I** ventral view **J** dorsal view **K** ventral view. Scale bars: 0.5 mm.

**Figure 3.**
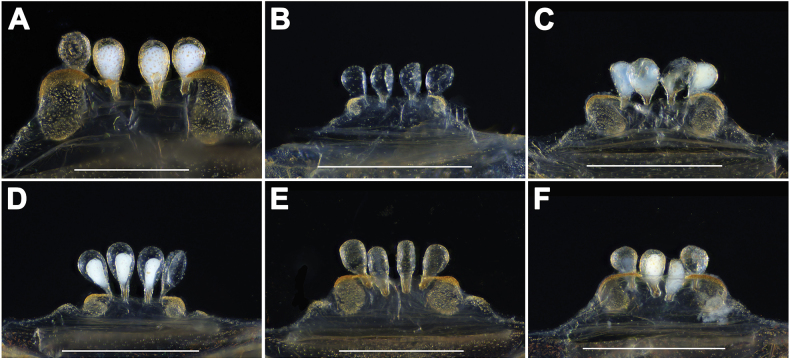
Female genital anatomy of *Atypusdawei* sp. nov. **A**–**F** vulva, dorsal view **A** ATY-2023-212 **B** ATY-2022-002 **C** ATY-2023-181 **D** ATY-2023-192 **E** ATY-2023-204 **F** ATY-2023-209. Scale bars: 0.5 mm.

The male palp of *A.dawei* sp. nov. resembles that of *A.flexus* Zhu, Zhang, Song & Qu, 2006 by having the embolus with thick, slightly curved base, but it can be distinguished from the latter by the larger, narrower conductor in prolateral view (Fig. 2G), and from that of *A.liui* sp. nov. by slightly curved and thicker base of the embolus, parallel to conductor (Fig. 2J, K) and by the smaller conductor (Fig. 2G–K). Female genitalia of *A.dawei* sp. nov. resemble those of *A.suiningensis* Zhang, 1985 and *A.liui* sp. nov. in having almost two equal-sized pairs of receptacula, but the new species can be distinguished from *A.suiningensis* by its nearly rectangular genital atrium, and from *A.liui* sp. nov. by the shorter stalks (Fig. 3A–F).

##### Description.

**Male** (holotype; Fig. 1F). TL (including chelicerae) 10.07. CL 3.82, CW 3.87, AL 3.41, AW 2.12. Carapace reddish brown. Fovea transverse with radial grooves, occupying about 1/7 of carapace width at that point (Fig. 2A). Eye diameter: AME 0.28, ALE 0.19, PME 0.16, PLE 0.07, AME–AME 0.10, AME–ALE 0.14, PME–PME 0.62, PME–PLE 0.05. MOA 0.50, front width 0.66, back width 0.88. Labium wider than long. Sternum yellowish brown, 2.49 long, 2.62 wide. Sigilla deeply imprinted, first pair close to anterior margin of sternum; posterior pair much larger and oval (Fig. 2B). Chelicerae reddish brown, with 12 teeth in a single row (Fig. 2C). Abdomen blackish brown with dorsal scutum light yellow. Legs slender and light yellow. Palpal femur with shallow furrow. Only femur I with granular texture on the prolateral side. Metatarsus IV with two dorsal spines. Leg measurements: leg I 13.15 (4.15, 1.58, 2.25, 3.10, 2.07), leg II 11.06 (3.45, 1.54, 1.74, 2.55, 1.78), leg III 10.00 (2.73, 1.39, 1.52, 2.48, 1.88), leg IV 13.46 (3.69, 1.52, 2.13, 3.58, 2.54).

***Palp***. Half-rounded conductor with an excurved distal edge (Fig. 2I–K); thick base of embolus slightly curved, and embolus parallel to conductor (Fig. 2G–K).

**Female** (ATY-2023-212; Fig. 1E). TL (including chelicerae) 16.27. CL 4.92, CW 4.86, AL 7.67, AW 5.13. Carapace reddish brown. Fovea transverse, occupying about 1/7 of carapace width at that point (Fig. 2D). Eye region black. Eye diameters: AME 0.24, ALE 0.20, PME 0.21, PLE 0.16, AME–AME 0.26, AME–ALE 0.24, PME–PME 0.91, PME–PLE 0.07. MOA 0.51, front width 0.70, back width 1.47. Sternum orange-brown, 4.34 long, 3.86 width, with dense covering of hairs. Sigilla larger than that in male, second pair smallest, fourth pair largest (Fig. 2E). Chelicerae reddish brown, with 14 teeth in a single row (Fig. 2F). Abdomen dark brown in oval, with an oval dorsal scutum in the anterior part. ALS 0.62 long, PMS 1.12 long, PLS four-segments as follows: basal 0.77, median 0.81, subapical 0.96, apical 1.09. Legs yellowish brown, with a dense covering of long hairs; all metatarsus with dorsal spines; metatarsus IV with six dorsal spines. Leg measurements: Leg I 11.35 (4.19, 2.04,1.74, 2.08, 1.30), Leg II 9.02 (2.94, 2.06, 1.35, 1.58, 1.09), Leg III 7.36 (2.04, 1.66, 1.19, 1.53, 0.94), Leg IV 10.39 (3.62, 1.75, 1.52, 2.20, 1.30).

***Vulva***. Genital atrium short, wider than long, with oval lateral pore patches; two pairs of pyriform receptacula similar in size, with short stalks (Fig. 3A–F).

##### Variation.

Females exhibit variation in body size and the number of cheliceral teeth. Measurements for females (*N* = 12) are as follows: TL 8.92–16.72, CL 3.13–4.92, CW 2.88–4.86, AL 3.61–7.67, AW 2.51–5.13. The number of cheliceral teeth ranges from 10 to 14 (*N* = 12). In addition, the female genitalia show intraspecific variation. The middle pair of receptacula are positioned either on the ventral side of the genitalia atrium (Fig. 3A, E, F) or near the anterior margin of the genitalia atrium (Fig. 3B–D).

##### Etymology.

The species epithet, a noun in apposition, refers to the type locality.

##### Distribution.

China (Anhui, Hunan, Jiangxi).

#### ﻿ Atypus liui

Taxon classificationAnimaliaAraneaeAtypidae

Li & Xu sp. nov.

809C9C2A-59B3-5C8B-B069-584A65C71B5F

https://zoobank.org/E20CF3E2-6D7E-47AF-A6C9-1BC73F9E67E2

Figs 4 , 5

##### Type material.

***Holotype***: China • ♂; Hunan Province, Linxiang City, Xiacaojiachong Village; 29.50°N, 113.35°E; alt. 67 m; 24 June 2018; D.Q. Li, F.X. Liu, X. Xu, D. Li leg.; YY-2018-001M.

***Paratypes***: China • 1♂6♀♀; same data as for holotype; YY-2018-001F–006 – Anhui Province • 1♀; Liuan City, Mount Mei; 31.71°N, 115.93°E; alt. 88 m; 15 June 2017; F.X. Liu, H. Liu leg.; AH-2017-023F – Guangxi Zhuang Autonomous Region • 2♀♀; Guilin City, Yongfu County, Yongfu Town, Jingmen Village; 25.01°N, 109.96°E; alt.117 m; 6 August 2024; X. Xu, Y.X. Li, Y.C. Xiong, S.S. Han leg.; ATY-2024-023, 024 – Guizhou Province • 2♂♂18♀♀; Qiandongnan Miao and Dong Autonomous Prefecture, Danzhai County, Xingren Town, Wudi Village; 26.24°N, 107.76°E; alt. 872 m; 8 August 2023; ATY-2023-095A–104D • 4♀♀; Qiannan Buyi and Miao Autonomous Prefecture, Duyun City, Yundong Town, Bagu Village; 26.24°N, 107.69°E; alt. 851 m; 8 August 2023; ATY-2023-089, 092B–094 • 8♀♀; Qiannan Buyi and Miao Autonomous Prefecture, Guiding County, Changming Town, Muyang Village; 26.27°N, 107.22°E; alt. 1089 m; 7 August 2023; ATY-2023-073, 074, 076, 077, 082A–082D • 1♀; Qiandongnan Miao and Dong Autonomous Prefecture, Danzhai County, Xingren Town, Machewan Village; 26.29°N, 107.80°E; alt. 887 m; 8 August 2023; X. Xu, Y.X. Li, Y.C. Xiong, S.S. Han leg.; ATY-2023-105 • 1♂9♀♀; Qiandongnan Miao and Dong Autonomous Prefecture, Sansui County, Kongjiawan Village; 26.94°N, 108.94°E; alt. 660 m; 16 August 2018; AT-2018-050–058 • 1♀; Qiandongnan Miao and Dong Autonomous Prefecture, Cengong County, Xiajiaao Village; 27.46°N, 108.83°E; alt. 556 m; 17 August 2018; AT-2018-060 • 7♀♀; Tongren City, Jiangkou County, Fanjingshan, Huangjiaba Village; 27.86°N, 108.78°E; alt. 547 m; 18 August 2018; F.X. Liu, D. Li, X. Xu, D.Q. Li, L. Yu leg.; AT-2018-064, 066–071 • 1♀; Tongren City, Yanhe Tujia Autonomous County, Heishui Town, Cuantang Village; 28.60°N, 108.43°E; alt. 721 m; 11 September 2021; ATY-2021-014 • 7♀♀; Zunyi City, Fenggang County, Yong’an Town, Zhengjiawan Village; 28.10°N, 107.59°E; alt. 939 m; 13 September 2021; ATY-2021-015–019, 021, 022 • 1♀; Zunyi City, Suiyang County, Wenquan Town, Shuanghe Village, Tianjiawan; 28.24°N, 107.29°E; alt. 966 m; 14 September 2021; ATY-2021-023 • 13♀♀; Zunyi City, Meitan County, Huangjiaba Town, Niuchang Village, Zhujiawan; 27.70°N, 107.40°E; alt. 802 m; 15 September 2021; ATY-2021-040–048D • 2♀♀; Zunyi City, Suiyang County, Tiantai Forest Park; 27.96°N, 107.18°E; alt. 955 m; 15 September 2021; X. Xu, Z.Y. Chen, Y. Zhan, Y. Zhang leg.; ATY-2021-029, 030 – Henan Province • 3♀♀; Dengfeng City, Shaolin Temple; 34.49°N, 112.93°E; alt. 704–843 m; 22 August 2016; LIF-2016-334, 336, 337 • 11♀♀; Xinxiang City, Baligou Scenic Spot, Tianhe Waterfall; 35.59°N, 113.53°E; alt. 679–758 m; 20 August 2016; F.X. Liu, Z.T. Zhang, H. Liu, F. Li leg.; LIF-2016-312–321-1 – Hubei Province • 3♀♀; Enshi City, Phoenix Forest Park; 30.28°N, 109.48°E; alt. 450–470 m; 27 March 2016; F.X. Liu, F. Li leg.; LIF-2016-045, 047, 049 • 3♀♀; Enshi Tujia and Miao Autonomous Prefecture, Hefeng County, Taiping Town, Zhongping Village; 29.79°N, 109.87°E; alt. 959 m; 13 August 2022; ATY-2022-047, 048A, 048D • 2♀♀; Enshi Tujia and Miao Autonomous Prefecture, Hefeng County, Yanzi Town, Shilongdong Village; 29.93°N, 110.16°E; alt. 1188 m; 13 August 2022; ATY-2022-045, 046 • 5♀♀; Enshi Tujia and Miao Autonomous Prefecture, Laifeng County, Sanhu Town, Datang Village; 29.58°N, 109.32°E; alt. 638 m; 14 August 2022; ATY-2022-049–050B, 050D • 2♀♀; Enshi Tujia and Miao Autonomous Prefecture, Xianfeng County, Qujiang Town, Gaopo Village; 29.60°N, 109.02°E; alt. 857 m; 14 August 2022; ATY-2022-051, 051B • 2♀♀; Yichang City, Wufeng Tujia Autonomous County, Changleping Town, Dawan Village; 30.19°N, 110.71°E; alt. 903 m; 12 August 2022; ATY-2022-024, 025 • 9♀♀; Yichang City, Wufeng Tujia Autonomous County; 30.16°N, 110.77°E; alt. 1076–1083 m; 12 August 2022; ATY-2022-015–021A, 021C • 9♀♀; Yichang City, Wufeng Tujia Autonomous County, Wantan Town, Honglie Village; 30.02°N, 110.41°E; alt. 1110 m; 13 August 2022; ATY-2022-026–028, 031–035A • 7♀♀; Yichang City, Wufeng Tujia Autonomous County, Wantan Town; 30.05°N, 110.36°E; alt. 1248 m; 13 August 2022; X. Xu, Y. Zhan, Y. Zhang leg.; ATY-2022-037–042, 044 • 4♀♀; Jianshi County; 30.57°N, 109.72°E; alt. 680 m; 7 October 2018; HB-2018-002, 004, 007, 010 • 2♀♀; Lichuan City, Tenglong Cave; 30.34°N, 108.98°E; alt. 1070 m; 6 October 2018; F.X. Liu, Z.C. Li leg.; HB-2018-003, 009 • 1♀; Lichuan City, Longtan Village; 30.30°N, 108.90°E; alt. 1092 m; 26 March 2016; F.X. Liu, F. Li leg.; LIF-2016-031 • 1♂2♀♀; Xianning City, Maqiao Town; 29.80°N, 114.36°E; 3 June 2017; F.X. Liu, C. Zeng leg.; XN-2017-001–003 • 1♀; Xianning City, Tingsi Town; 29.82°N, 114.17°E; alt. 73 m; 12 March 2016; LIF-2016-016 • 3♂♂6♀♀; Xianning City, Taiyi Avenue, Jinchahua Farm; 29.80°N, 114.32°E; alt. 27 m; 13 July 2017; F. Li leg.; Jch-2017-001–010 – Hunan Province • 1♀; Loudi City, Louxing District, Yijiazhou Village; 27.69°N, 111.95°E; alt. 130 m; 9 August 2018; AT-2018-017 • 5♀♀; Huaihua City, Hongjiang City, Lingshang Village; 27.29°N, 110.40°E; alt. 836 m; 11 August 2018; AT-2018-025–028A • 2♀♀; Huaihua City, Hongjiang City, Xuefeng Town, Jiejiao Village; 27.29°N, 110.40°E; alt. 967 m; 11 August 2018; AT-2018-030C, 030D • 2♀♀; Changde City, Dingcheng District, Huayanxi; 28.73°N, 111.52°E; alt. 123 m; 21 August 2018; AT-2018-095, 095A • 4♀♀; Huaihua City, Yuanling County, Maopingtou Village; 28.46°N, 110.55°E; alt. 365 m; 21 August 2018; F.X. Liu, D. Li, X. Xu, D.Q. Li, L. Yu leg.; AT-2018-088, 091, 093, 094 • 7♀♀; Shimen County, Hupingshan Town, Sanhe Village; 30.09°N, 110.80°E; alt. 760 m; 25 October 2018; AT-2018-097–103 • 1♀; Hengyang City, Gouloufeng; 27.12°N, 112.63°E; alt. 660–800 m; 28 October 2018; F.X. Liu leg.; AT-2018-105 • 1♀; Changde City, Shimen County, Huping Mountain Scenic Area, Hupingshan Village; 30.11°N, 110.77°E; alt. 1060 m; 22 September 2019; ATY-2019-022 • 2♀♀; Zhangjiajie City, Zhangjiajie National Forest Park, 29.39°N, 110.49°E; alt. 1109–1154 m; 24 September 2019; X. Xu, D.Q. Li leg.; ATY-2019-031, 033 • 1♀; Liuyang City, Mount Dawei; 28.41°N, 114.06°E; alt. 700 m; 9 May 2021; X. Xu leg.; ATY-2021-002 • 2♀♀; Huaihua City, Xupu County, Nanhe Village; 28.17°N, 110.78°E; alt. 255 m; 7 September 2021; X. Xu, Z.Y. Chen, Y. Zhan, Y. Zhang leg.; ATY-2021-004, 005 • 1♀; Shaoyang City, Dongkou County, Gaosha Town, Yangsiqiao Village; 26.93°N, 110.62°E; alt. 330 m; 24 August 2022; X. Xu, Y. Zhan, Y. Zhang, Y.X. Li leg.; ATY-2022-077D • 1♀; Shaoyang City, Wugang City, Caoqi Village; 26.78°N, 110.69°E; alt. 321 m; 11 August 2023; X. Xu, Y.X. Li, Y.C. Xiong, S.S. Han leg.; ATY-2023-153 • 1♀; Hengyang City, Hengyang County, Guanshi Town; 26.90°N, 112.07°E; alt. 183 m; 4 August 2024; X. Xu, Y.X. Li, Y.C. Xiong, S.S. Han leg.; ATY-2024-009A – Jiangxi Province • 1♂23♀♀; Jiujiang City, Mount Lu; 29.55°N, 115.98°E; alt. 1044–1010 m; 22 May 2017 and 24 May 2018; F.X. Liu, H. Liu, Z.C. Li leg.; LS-2017-001–004, 007–010, LS-2018-001–016M • 1♀; Jiujiang City, Lianxi District, Nanshan Park; 29.67°N, 115.99°E; alt. 50 m; 3 September 2023; ATY-2023-216 • 1♀; Pingxiang City, Anyuan District, Gaokeng Town, Luojiatang Village; 27.64°N, 113.94°E; alt. 182 m; 26 August 2023; ATY-2023-167 • 2♀♀; Xinyu City, Yushui District, Baoshi Park; 27.80°N, 114.93°E; alt. 79 m; 27 August 2023; X. Xu, Y. Zhang, Y.X. Li, J.Y. Yuan leg.; ATY-2023-170, 171B.

##### Diagnosis.

**Figure 4.**
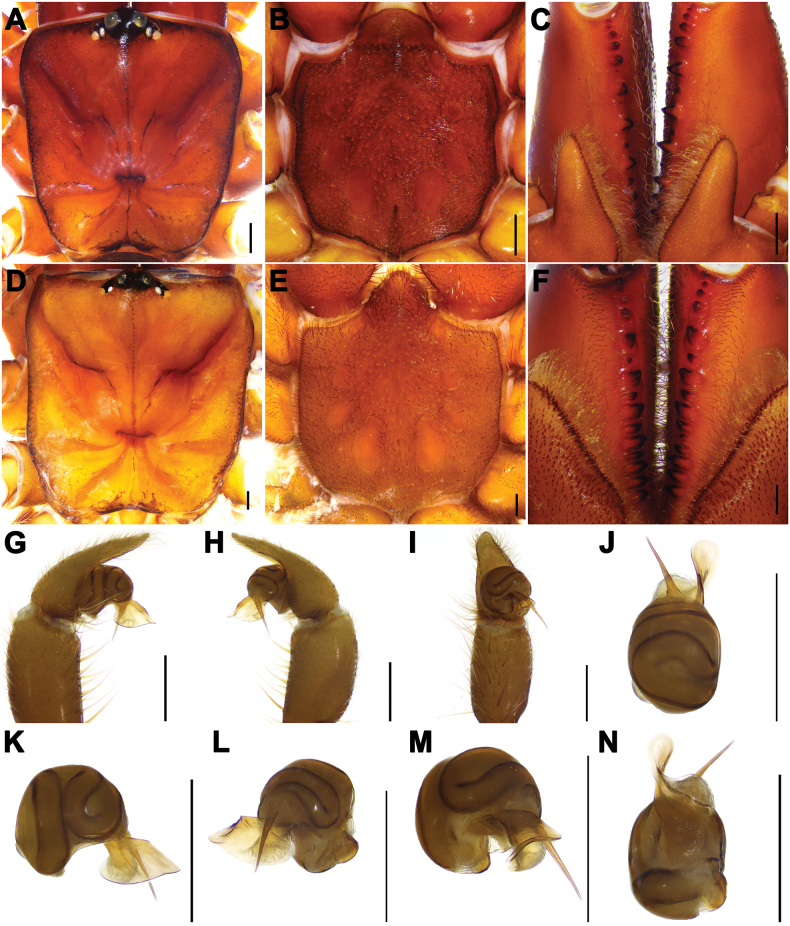
Male and female somatic structure and male genital anatomy of *Atypusliui* sp. nov. **A–C** male (YY-2018-001M) **D–F** female (YY-2018-001F) **A, D** carapace **B, E** sternum **C, F** chelicerae **G–N** left male palp **G–I** YY-2018-001M **J–N** ATY-2023-102M **G, K** prolateral view **H, L** retrolateral view **I, M** ventral view **J** dorsal view **N** ventral view. Scale bars: 0.5 mm.

**Figure 5.**
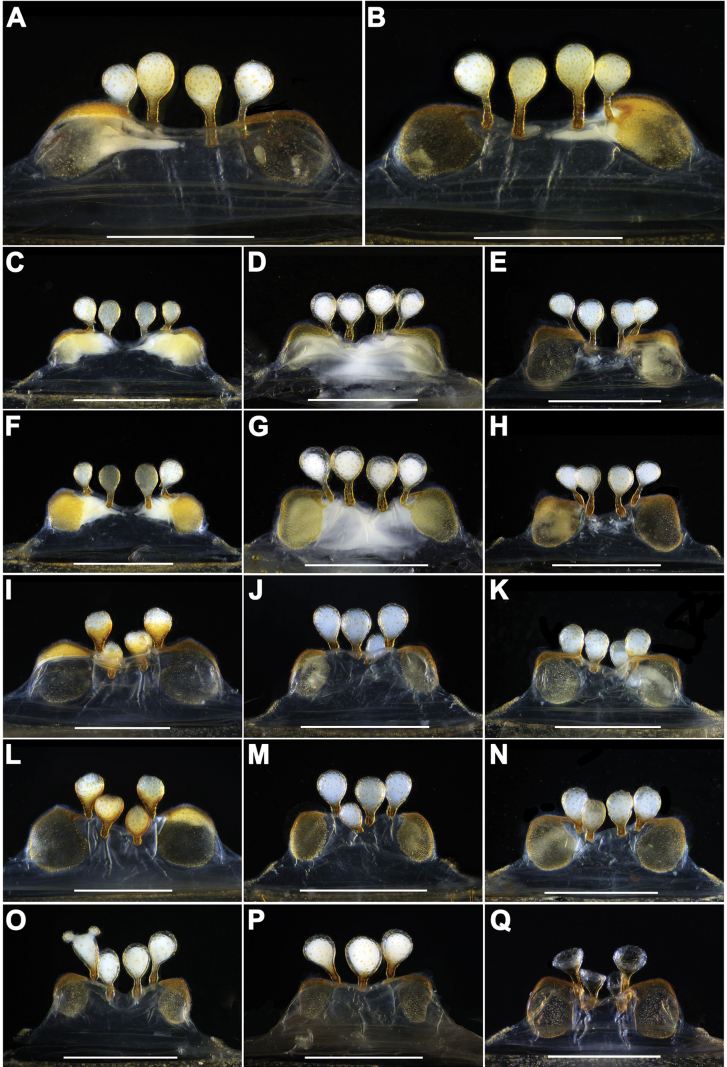
Female genital anatomy of *Atypusliui* sp. nov. **A**, **C–E, I–K, O–Q** vulva, dorsal view **B, F–H**, **L–N** vulva, ventral view **A, B** YY-2018-001F **C, F** AT-2018-017 **D, G** AT 2018-064 **E, H** ATY-2022-024 **I, L** YY-2018-005F **J, M** AT-2018-053 **K, N** ATY-2022-050B **O** ATY-2023-082A **P** ATY-2023-082C **Q** ATY-2021-048C. Scale bars: 0.5 mm.

The male palp of *A.liui* sp. nov. resembles those of *A.heterothecus* Zhang, 1985 and *A.largosaccatus* Zhu, Zhang, Song & Qu, 2006 in having the apex of the embolus pointing toward the tibia, but the new species can be distinguished from the others by its slightly longer embolus and slightly larger conductor (Fig. 4J–N), and by the less excurved distal edge of the embolus (Fig. 4K–M). Female genitalia of *A.liui* sp. nov. resemble those of *A.largosaccatus* by the similarly sized receptacula with slightly long stalks, but the new species can be distinguished from the latter by the genital atrium, which has larger, paired oval lateral pore patches (Fig. 5), and from *A.karschi* Dönitz, 1887 by the slightly longer stalks (Fig. 5A–H) with incrassate bases (Fig. 5A–Q).

##### Description.

**Male (*holotype*)**. TL (including chelicerae) 11.33. CL 4.10, CW 3.30, AL 4.42, AW 2.74. Carapace smooth and reddish brown. Fovea transverse with radial grooves, occupying about 1/7 of carapace width at that point (Fig. 4A). Eye diameter: AME 0.24, ALE 0.23, PME 0.15, PLE 0.18, AME–AME 0.23, AME–ALE 0.11, PME–PME 0.65, PME–PLE 0.02. MOA 0.50, front width 0.70, back width 1.19. Labium wider than long. Chelicerae reddish brown, with 13 teeth in a single row on promargin, distal two teeth obvious small (Fig. 4C). Sternum dark reddish brown, 2.47 long, 2.36 wide. Sigilla deeply imprinted, with first pair attaching to anterior margin of sternum; second pair rounded, third and fourth pairs oval (Fig. 4B). Abdomen light brown and oval, with a nearly oval dorsal scutum. Spinnerets six: ALS 0.36 long, PMS 0.71 long, PLS with four segments: basal 0.51, median 0.49, subapical 0.46, apical 0.42. Palpal femur lacking furrow in prolateral side. Legs slender and light yellow. Granular texture only on the prolateral side of femur I. Metatarsus IV with one dorsal spine. Leg measurements: Leg I 12.00 (3.91, 1.67, 2.09, 2.60, 1.73), Leg II 9.91 (2.84, 1.50, 1.70, 2.30, 1.57), Leg III 9.62 (2.55, 1.29, 1.49, 2.46, 1.83), Leg IV 12.40 (3.50, 1.43, 2.13, 3.30, 2.04).

***Palp*.** Conductor with an excurved distal edge (Fig. 4I, M, N); long embolus straight and separated from conductor distally (Fig. 4H, J).

**Female** (YY-2018-001F). TL (including chelicerae) 19.38. CL 5.82, CW 5.61, AL 9.26, AW 6.95. Carapace smooth and yellowish brown, with black margin. Fovea transverse, occupying about 1/5 of carapace width at that point (Fig. 4D). Eye diameters: AME 0.27, ALE 0.27, PME 0.17, PLE 0.16, AME–AME 0.40, AME–ALE 0.25, PME–PME 1.13, PME–PLE 0.09. MOA 0.60, front width 0.87, back width 1.82. Chelicerae reddish brown, with 15 teeth on promargin in a single row (Fig. 4F). Sternum orange-brown, with a dense covering of short hairs, 4.37 long, 4.44 width. Labium wider than long (Fig. 4E). Abdomen dark brown, with an obvious dorsal scutum. Six spinnerets: ALS 0.59 long, PMS 1.33 long, PLS with four segments: basal 1.02, median 0.97, subapical 0.83, apical 0.76. Legs orange-brown, with a dense covering of long hairs and spines; all metatarsus with dorsal spines; metatarsus IV with 10 dorsal spines. Leg measurements: Leg I 13.51 (5.05, 2.45, 2.09, 2.44, 1.48), Leg II 11.25 (3.93, 2.39, 1.56, 1.97, 1.40), Leg III 9.69 (3.16, 2.10, 1.43, 1.93, 1.07), Leg IV 11.7 (3.87, 2.14, 1.88, 2.70, 1.11).

***Vulva*.** Genital atrium short, with a pair of large, oval lateral pore patches; two pairs of bulbous receptacula, with long and incrassate stalks; two pairs of similarly sized receptacula attached to the anterior margin or slightly ventral side of genital atrium (Fig. 5A–Q).

##### Variation.

Males and females vary in body size and the number of cheliceral teeth. Measurements for males are as follows (*N* = 9): TL 9.53–15.93, CL 3.04–4.66, CW 2.91–4.96, AL 3.31–5.91, AW 2.22–4.45. The number of cheliceral teeth ranges from 10 to 16 (*N* = 9). Measurements for females (*N* = 210) are as follows: TL 8.09–22.26, CL 2.52–6.40, CW 2.8–6.88, AL 2.69–11.60, AW 2.59–8.77. The number of cheliceral teeth varies from 6 to 21 (*N* = 210). In addition, the female genitalia exhibit intraspecific variations. Two pairs of receptacula exhibit either long stalks (Fig. 5A–H, O, Q) or slightly short stalks (Fig. 5I–N, P). Two pairs of receptacula are positioned on the anterior margin of the genital atrium (Fig. 5A–H, J–K, M–N, P) or the middle pair are situated on the ventral side of the genital atrium (Fig. 5I, L, O, Q). The vulva typically possesses either four receptacula (Fig. 5A–O, Q) or three receptacula (Fig. 5P), or with one of the lateral receptacula having two small protuberances on the top (Fig. 5O).

##### Etymology.

The specific name is dedicated to Mr Fengxiang Liu for his kind instructions on the collection.

##### Distribution.

China (Anhui, Guangxi, Guizhou, Henan, Hubei, Hunan, Jiangxi).

## Supplementary Material

XML Treatment for Atypus dawei

XML Treatment for Atypus liui

## ﻿Additional information

### Author ORCIDs

Xin Xu https://orcid.org/0000-0001-5632-6622

### Data availability

All of the data that support the findings of this study are available in the main text.
